# Semi-Supervised KPCA-Based Monitoring Techniques for Detecting COVID-19 Infection through Blood Tests

**DOI:** 10.3390/diagnostics13081466

**Published:** 2023-04-18

**Authors:** Fouzi Harrou, Abdelkader Dairi, Abdelhakim Dorbane, Farid Kadri, Ying Sun

**Affiliations:** 1 Computer, Electrical and Mathematical Sciences and Engineering (CEMSE) Division, King Abdullah University of Science and Technology (KAUST), Thuwal 23955-6900, Saudi Arabia; 2 Computer Science Department, University of Science and Technology of Oran-Mohamed Boudiaf (USTO-MB), El Mnaouar, BP 1505, Oran 31000, Algeria; abdelkader.dairi@univ-usto.dz; 3 Smart Structures Laboratory (SSL), Department of Mechanical Engineering, Belhadj Bouchaib University of Ain Temouchent, Ain Temouchent 46000, Algeria; 4 Aeroline DATA & CET, Agence 1031, Sopra Steria Group, 31770 Colomiers, France

**Keywords:** COVID-19, routine blood tests, kernel PCA, semi-supervised anomaly detection, data-driven

## Abstract

This study introduces a new method for identifying COVID-19 infections using blood test data as part of an anomaly detection problem by combining the kernel principal component analysis (KPCA) and one-class support vector machine (OCSVM). This approach aims to differentiate healthy individuals from those infected with COVID-19 using blood test samples. The KPCA model is used to identify nonlinear patterns in the data, and the OCSVM is used to detect abnormal features. This approach is semi-supervised as it uses unlabeled data during training and only requires data from healthy cases. The method’s performance was tested using two sets of blood test samples from hospitals in Brazil and Italy. Compared to other semi-supervised models, such as KPCA-based isolation forest (iForest), local outlier factor (LOF), elliptical envelope (EE) schemes, independent component analysis (ICA), and PCA-based OCSVM, the proposed KPCA-OSVM approach achieved enhanced discrimination performance for detecting potential COVID-19 infections. For the two COVID-19 blood test datasets that were considered, the proposed approach attained an AUC (area under the receiver operating characteristic curve) of 0.99, indicating a high accuracy level in distinguishing between positive and negative samples based on the test results. The study suggests that this approach is a promising solution for detecting COVID-19 infections without labeled data.

## 1. Introduction

Detection of contaminated cases with COVID-19 is crucial for controlling the spread of the virus [1]. When contaminated cases are identified, they can be isolated and treated, preventing them from spreading the virus to others. This is particularly important for asymptomatic carriers who may unknowingly spread the virus. Early detection also allows for contact tracing, where individuals who have been in close contact with the infected person can be identified and tested, further reducing the spread of the virus. Detection of contaminated cases can also inform public health decisions such as implementing quarantine measures, closing public spaces, and adjusting social distancing guidelines. It also helps to allocate resources, such as medical supplies and hospital beds, more effectively. Additionally, detecting contaminated cases can help to identify outbreaks and hotspots, allowing health officials to target their efforts to the areas that need it most, slowing the spread of the virus and preventing overwhelmed healthcare systems [2]. In short, detecting contaminated cases is essential for controlling the spread of COVID-19. It helps to identify and isolate infected individuals, trace contacts, inform public health decisions, allocate resources, and target efforts to the areas that need it most [3].

The identification of infected individuals through accurate and efficient procedures is crucial for effectively controlling the spread of COVID-19. In recent times, various machine learning techniques that utilize routine blood tests have been proposed as a means to address the limitations of traditional real-time reverse transcription–polymerase chain reaction (RT-PCR) tests [4,5,6]. These machine learning-based strategies have the potential to provide faster and more accurate results, making them a valuable tool in the fight against COVID-19. However, it is important to note that the research and development of these AI-based COVID-19 detection methods are still ongoing, and more studies are needed to validate their accuracy and reliability before they can be implemented on a large scale. In [7], Wang et al. proposed a method for detecting whether people are wearing masks during the COVID-19 pandemic, using transfer learning and broad learning systems. They proposed a two-stage approach that involves detecting candidate regions where a mask may be worn using a transfer model; they used a broad learning system to verify if the detected regions were authentic facial masks. They also introduced a dataset for mask-wearing detection, which included 7804 realistic images. The results show that this approach achieves an overall accuracy of 97.32% for simple scenes and 91.13% for complex scenes, outperforming the compared methods. They suggest that the proposed method can help curb the spread of COVID-19 by detecting masked faces in public spaces, such as hospitals and airports. In [8], Sharma et al. proposed an eigenvalue decomposition of the Hankel matrix (EVDHM)-based autoregressive integrated moving average (ARIMA) model to predict the number of COVID-19 cases. They decomposed the data into subcomponents using EVDHM to reduce non-stationarity, applied ARIMA to forecast future values for each subcomponent, and combined the results to generate the final output values. They employed a genetic algorithm to obtain the optimal values of ARIMA parameters based on the minimum Akaike information criterion. This technique is applied to predict daily new COVID-19 cases in India, the USA, and Brazil, showing high effectiveness. In [9], Lam et al. used semi-supervised learning (SSL) techniques to develop and validate machine learning algorithms for predicting the development of acute respiratory distress syndrome (ARDS) in hospitalized patients [9]. The study employed a dataset of 29,127 encounters with patients admitted to seven US hospitals, and a recurrent neural network was utilized to predict ARDS development based on electronic health record data. The study’s outcomes indicate that SSL techniques can enhance the model performance, and unlabeled data can be used for predicting ARDS development in scenarios where labeled data are relatively inexpensive. The area under the receiver operating characteristic curve increases from 0.73 to 0.84 when using the labeled dataset. The paper concludes that unlabeled data can be valuable for enhancing the efficiency of machine learning models in predicting ARDS development.

In [10], W. Wu et al. presented a method for reconstructing high-quality computed tomography (CT) images in low-dose cases, based on a technique called tensor gradient L0-norm minimization (TGLM). They developed a TGLM strategy to reduce radiation dose by considering the 3D spatial information of CT images. The optimization of the TGLM model was carried out using the split Bregman technique, and its performance was subsequently evaluated using two COVID-19 patients as test cases. Results revealed that the TGLM model exhibits accurate detection performance in scenarios involving low-dose cases, and it effectively preserves image edges while minimizing sparse undersampling artifacts. Various studies have been conducted to detect COVID-19 infection using chest X-rays. For instance, Han et al. present a semi-supervised deep neural network method for improving the detection of COVID-19 using CT images in [11]. The method utilizes labeled and unlabeled CT images to enhance the accuracy and robustness of COVID-19 diagnosis. This approach achieves an overall accuracy of 99.83%, a sensitivity of 0.9286, a specificity of 0.9832, and a positive predictive value (PPV) of 0.9192 in distinguishing COVID-19 from non-COVID-19 CT images. Additionally, the method obtains 97.32% accuracy, 0.9971 sensitivity, 0.9598 specificity, and 0.9326 PPV in discriminating between COVID-19 and common pneumonia CT images. The results are consistent across different datasets, and the proposed method improves diagnostic accuracy and robustness without exhaustive labeling.

In [12], Khobahi et al. considered a deep learning-based technique for analyzing chest X-ray images to diagnose COVID-19. This method uses a semi-supervised learning approach based on autoencoders to extract infected legions in chest X-ray images. Then it utilizes a deep architecture to extract relevant features and train a classifier for automatic diagnosis. The results showed that this approach achieved an overall class average accuracy of 93.5%. Brunese et al. proposed a deep learning-based approach for the rapid and automatic detection of COVID-19 from X-rays in [13]. The approach consists of three phases: pneumonia detection, discrimination between COVID-19 and pneumonia, and localization of the COVID-19 presence in symptomatic areas of the X-ray. The method was evaluated on a dataset comprising 6523 chest X-rays from multiple institutions, and it demonstrated an average detection time of 2.5 s and an average accuracy of 0.97.

Recently, Dairi et al. in [14] proposed an approach for detecting COVID-19 infection using blood test samples. They addressed the problem of COVID-19 infection detection as an anomaly detection problem through the use of a semi-supervised deep hybrid model. The method combines the use of a variational autoencoder (VAE) deep learning model for feature extraction and a one-class support vector machine (OCSVM) for separating infected from non-infected COVID-19 individuals. The performance of the model was evaluated using blood test samples from hospitals in Brazil and Italy. The results indicate that the VAE-based OCSVM detector surpasses the other methods, such as generative adversarial networks (GANs) and restricted Boltzmann machines (RBMs), in terms of discrimination performance for detecting potential COVID-19 infections. Moreover, in [15], Alves et al. investigated machine learning techniques for COVID-19 diagnosis through routine blood tests, using a public dataset from a Brazilian hospital. The random forest (RF) performed the best among the classifiers tested. A decision tree explainer (DTX) was utilized for localized explanations to improve interpretability. A criteria graph was employed to aggregate these explanations and offer a comprehensive understanding of the results. The authors argue that this approach closely mimics the decision-making process of healthcare professionals, making it more practical for real-world use. The study findings indicate that simple blood tests can assist in identifying false positives/negatives in RT-PCR tests. In [16], AlJame et al. introduced an ensemble learning technique, named ERLX, for identifying COVID-19 from routine blood tests. This model incorporates three distinct classifiers (extra trees, random forest, and logistic regression) in the initial stage, and then utilizes a second-level classifier, known as extreme gradient boosting (XGBoost), to merge the predictions and enhance the performance. The data preparation process includes handling null values with KNNImputer, removing outliers with iForest, and balancing data distribution with SMOTE. The model’s interpretability is enhanced using the SHapley Additive exPlanations (SHAP) technique to report feature importance. The results from a publicly available dataset from the Albert Einstein Hospital in Brazil show that the ERLX model achieved an overall accuracy of 99.88%, an AUC of 99.38%, a sensitivity of 98.72%, and a specificity of 99.99%. This model is considered more robust and reliable than existing state-of-the-art models for early and rapid screening of COVID-19 patients. In [17], an approach called TESSOM (tree-based entropy-structured self-organizing maps) is proposed for identifying pertinent attributes in blood test examinations for COVID-19 diagnosis. Specifically, this approach uses self-organizing maps and an entropy calculation to create a hierarchical, semi-supervised, and explainable model. By enhancing investigating groups of cases with high levels of class overlap, the algorithm enables a simpler explanation of results to experts. The results of their experiments, which analyzed 2207 cases from three hospitals in Brazil, demonstrate that TESSOM improves COVID-19 case identification and offers a performance increase of 1.489% in multiple scenarios.

The study conducted by Brinati et al. in [18] aimed to create machine learning models for identifying COVID-19 using routine blood tests as an alternative to the standard RT-PCR test. The study utilized hematochemical values from routine blood exams of 279 patients admitted to a hospital with COVID-19 symptoms, out of which 177 were positive, and 102 were negative. Two machine learning models, known as the three-way random forest (TWRF) classifier and RF, were developed using demographic characteristics and a small set of routine blood tests. The performance of the models was comparable to the gold standard test, with an accuracy range of 82–86% and a sensitivity range of 92–95%. Additionally, these models outperformed traditional classifiers, such as decision trees, extremely randomized trees, KNN, logistic regression, and naive Bayes. Freitas Barbosa et al. [19] developed an intelligent system, called Heg.IA, to aid in the diagnosis of COVID-19 using blood test results. The system utilizes laboratory parameters obtained from hemogram and biochemical tests as input features and employs particle swarm optimization, evolutionary algorithms, and manual feature selection to identify the most important features.

Several machine learning models were evaluated, and the highest classification performance was achieved with an overall accuracy of 95.159%, a kappa index of 0.903, a sensitivity of 0.968, a precision of 0.938, and a specificity of 0.936. The best classifier was found to be the Bayes network. The authors suggest that this system could serve as a low-cost rapid test and is available for free use. The study by Aktar et al. in [20] aimed to determine how blood data from COVID-19 patients can be used to predict clinical outcomes. They used a combination of statistical methods, correlation analysis, and machine learning algorithms (such as RF, SVM, DT, and LGBM) to analyze clinical data from patients with known outcomes. They found that certain measurable clinical parameters in blood samples could distinguish between healthy individuals and those who are positive for COVID-19, and could also predict the severity of symptoms. The authors developed analytical methods with accuracy and precision scores above 90% for predicting disease severity. They concluded that their approach could be used to identify patients at high risk of mortality and optimize hospital resources for COVID-19 treatment.

It is worth noting that while many countries have progressed in controlling the spread of COVID-19, ongoing research into effective and efficient diagnostic methods is still necessary. The approach proposed in this study offers a promising solution that can be applied to future outbreaks, highlighting the study’s relevance and importance beyond the current COVID-19 pandemic. This study presents a new semi-supervised method for identifying COVID-19 infection using blood test data as part of an anomaly detection problem. The approach merges the capabilities of kernel principal component analysis (KPCA) with an unsupervised OCSVM to differentiate healthy from infected COVID-19 cases based on blood test samples. This method addresses traditional blood test limitations, such as cost and time consumption, by providing a flexible and data-driven approach to COVID-19 detection. KPCA is employed to identify nonlinear patterns in the data, while OCSVM is used to detect abnormal features. This approach is semi-supervised as it uses unlabeled data during the training process, and it only requires data from healthy cases to construct the model. This approach also has the advantage of detecting anomalies in the data, potentially identifying infected individuals even if they are asymptomatic or have atypical symptoms. Therefore, this study’s approach has practical implications for public health efforts to control and prevent the spread of COVID-19. The model’s performance was tested on two sets of blood test samples from hospitals in Brazil and Italy. Compared to other semi-supervised models, such as KPCA-based isolation forest (iForest), local outlier factor (LOF), and elliptical envelope (EE) schemes, independent component analysis (ICA), and PCA-based monitoring techniques, the proposed KPCA-OSVM approach delivers good discrimination capability for detecting potential COVID-19 infections.

In Section 2, we provide an overview of the OCSVM and KPCA algorithms, as well as the proposed semi-supervised KPCA-based OCSVM detector. Additionally, Section 2 offers a brief review of the benchmark methods used in this study, including PCA, ICA, iForest, LOF, and EE. In Section 3, we evaluate the performance of the proposed approach using two publicly available datasets. Finally, in the last section, we summarize this research and offer future research directions.

## 2. Materials and Methods

In this section, we provide an overview of all of the necessary components for this study. This includes a brief explanation of OCSVM, KPCA, the proposed KPCA-OCSVM method, as well as the benchmark methods used, such as PCA, ICA, iForest, LOF, and EE.

### 2.1. Kernel PCA Model

In this study, the KPCA algorithm, an extension of linear PCA, is used to uncover nonlinear relationships among process variables to improve fault detection performance. The KPCA algorithm transforms the input space into a higher-dimensional feature space via a nonlinear mapping function, using kernel tricks on the data. This approach allows for the discovery of nonlinear patterns in the data that may not be apparent in the original space, making it a powerful tool for dimensionality reduction and feature extraction. The KPCA algorithm is then used to feed a one-class SVM scheme for anomaly detection. The one-class SVM models the normal behavior of the system and detects any deviation from this normal behavior as an anomaly, thus improving performance over traditional linear PCA-based monitoring methods. By using KPCA to extract relevant features, this approach reduces the high computation cost associated with nonlinear optimization compared to other nonlinear methods. Additionally, the use of kernel tricks allows the KPCA algorithm to handle nonlinearity without the need for explicit computation of nonlinear functions, which can be computationally expensive. Furthermore, the use of KPCA with one-class SVM also reduces the need for labeled data and is less sensitive to the choice of the kernel function, making it a more robust method for anomaly detection.

In this study, the original training dataset, denoted as $x1,x2\cdots ,xn\in R^{m}$, where *n* is the number of samples and *m* is the number of process variables, is used to construct a feature space through a nonlinear mapping function $\Phi \left(•\right):R^{m}\to F^{h}$. The dimension of the feature space is represented by *h*, a large positive integer. Similarly to PCA, the covariance matrix in the feature space F, denoted as ΣF, is calculated by using this nonlinear mapping function.

(1) $$\Sigma_{F}=\frac{1}{n}\sum_{i=1}^{n}\left[\Phi \left(x_{i}\right)-m_{\Phi }\right]{\left[\Phi \left(x_{i}\right)-m_{\Phi }\right]}^{T},$$

The sample mean in the feature space, denoted as mΦ, is calculated by summing the mapped points and dividing by the number of samples. Each mapped point is then centered by subtracting the sample mean from it and represented as $\bar{\Phi }\left(x_{i}\right)$. The principal components are found by solving the eigenvalue decomposition problem in the feature space.

(2) $$\lambda v=\Sigma_{F}v=\frac{1}{n}\sum_{i=1}^{n}\left[\bar{\Phi }{\left(x_{i}\right)}^{T}v\right]\bar{\Phi }\left(x_{i}\right),$$

The eigenvalue and eigenvector of the covariance matrix ΣF in the feature space are represented by λ and v, respectively. By multiplying $\bar{\Phi }\left(xj\right)$ with Equation (2), the kernel matrix K or $\bar{K}$ of dimension n×n is defined as:

(3) $$K\left(x_{i},x_{j}\right)=\Phi {\left(x_{i}\right)}^{T}\Phi \left(x_{j}\right),$$

(4) $$\bar{K}\left(x_{i},x_{j}\right)=\bar{\Phi }{\left(x_{i}\right)}^{T}\bar{\Phi }\left(x_{j}\right),$$

(5) $$\bar{K}=K-KE-EK+EKE,$$

in conjunction with $\alpha \in R^{n}$ to represent the kernel principal components by the feature space training samples, fulfilling the equation:

(6) $$v=\sum_{i=1}^{n}\alpha_{i}\bar{\Phi }\left(x_{i}\right).$$

The problem of eigenvalue decomposition was then transformed, as described in [21],

(7) $$n\lambda \alpha =\bar{K}\alpha .$$

The nonlinear patterns in the data can be captured by the eigenvectors determined in the feature space F, also known as kernel principal components. The number of eigenvectors is equal to the number of samples, which is typically much higher than the number of linear principal components that can be obtained using traditional PCA.

### 2.2. One-Class SVM

The OCSVM is a popular technique used for anomaly detection, which identifies abnormal data instances in a dataset. OCSVM is a type of SVM that learns a decision boundary that separates the normal data from the rest, and is considered anomalous [22]. OCSVM is a semi-supervised learning algorithm used for detecting anomalies in a given dataset. Unlike traditional supervised learning methods, OCSVM does not require labeled data for model construction and is trained using only “normal” observations [23]. The basic idea behind OCSVM is to identify a boundary that maximizes the gap between the origin and the normal observations in the training data points in their original space. This boundary is then utilized to classify new test data as comparable or distinct from the training data [24]. Importantly, OCSVM creates a decision boundary around the normal data points, which can then be used to identify the anomalous data points. In conclusion, OCSVM is a powerful and flexible tool for anomaly detection, particularly when the training data are limited to only one class [25]. With its ability to identify abnormal instances, it has the potential to be applied in a wide range of fields, from healthcare to cybersecurity.

The goal of OCSVM is to find a hyperplane that maximizes the margin between the hyperplane and the closest normal observations while separating the normal observations from the origin. This ensures that the hyperplane is as far away from the anomalous observations as possible. After determining the hyperplane, new observations can be classified as normal or anomalous based on which side of the hyperplane they fall on. This is accomplished by solving the optimization problem represented by Equation (8).

(8) $$\min_{\omega \gamma \rho }\left(\frac{1}{2}\omega^{T}\omega -\rho +\frac{1}{\upsilon l}\sum_{i=1}^{l}\gamma_{i}\right),$$

Subject to:

ω.Ψ(x)>ρ−γ.

As shown in (8), the OCSVM scheme uses a weight vector (ω), a regularization term (υ), a slack variable (γ), and an offset term (ρ) to calculate the decision boundary. The regularization term (υ) is used to avoid overfitting. The algorithm incorporates a slack variable, denoted as γ, to account for observations that fall outside the decision boundary during the training stage. Additionally, an offset term, represented by ρ, is used to determine the distance between the origin and the mapped samples in the feature space. The decision function of the OCSVM algorithm, given by F, returns −1 for an anomaly and 1 for a typical data point based on the hyperplane.

(9) $$F\left(x\right)=\mathrm{sign}\left(\omega .\;\Psi (x)-\rho \right).$$

The function Ψ is used to transform the original data samples into a higher-dimensional feature space. The hyperplane, defined by the term $\frac{\rho }{∥\omega ∥}$, is the Euclidean distance from the origin to the support vector point. The goal is to maximize this term. The OCSVM algorithm solves a quadratic optimization problem as stated in Equation (10).

(10) $$\min_{\omega \gamma \rho }\left(\frac{{∥\omega ∥}^{2}}{2}-\rho +\frac{1}{\upsilon l}\sum_{i=1}^{l}\gamma_{i}\right).$$

The OCSVM algorithm seeks to maximize the margin between the origin and the mapped data in the feature space [26]. This is achieved by maximizing the term $\frac{{∥\omega ∥}^{2}}{2}-\rho$ in the objective function and minimizing the average of the slack variables γ. There are several kernel functions available in the literature that can be used with OCSVM. The most commonly used ones are [27]:

- Radial basis function (RBF) kernel:
  (11) $$K\left(x,x^{\prime }\right)=\langle \Psi \left(x\right),\Psi \left(x^{\prime }\right)\rangle =e^{\left(\alpha ∥x-x^{\prime }{∥}^{2}\right)},$$
  with the dissimilarity measure being the square of the distance between two data points and the kernel parameter represented by α.
- Linear kernel:
  (12) $$K\left(x,x^{\prime }\right)=x^{T}\cdot x^{\prime }.$$
- Polynomial kernel:
  (13) $$K\left(x,x^{\prime }\right)={\left(\gamma \cdot x^{T}\cdot x^{\prime }+r\right)}^{d},$$
  where γ is a scaling parameter, *r* is a constant term, and *d* is the degree of the polynomial.
- Sigmoid kernel:
  (14) $$K\left(x,x^{\prime }\right)=\tanh\left(\gamma \cdot x^{T}\cdot x^{\prime }+r\right),$$
  where γ and *r* are scaling and constant parameters, respectively.

Where x and $x^{\prime }$ are the input feature vectors, and $K(x,x^{\prime })$ is the kernel function that measures the similarity between two input vectors in a higher-dimensional feature space. The performance of OCSVM with different kernel functions depends on the nature of the data and the problem being solved. The RBF kernel generally performs well for most datasets because it can model complex decision boundaries. However, the linear kernel may be more appropriate if the data are linearly separable.

### 2.3. The Proposed KPCA-OCSVM Anomaly Detection Approach

**Figure 1:**
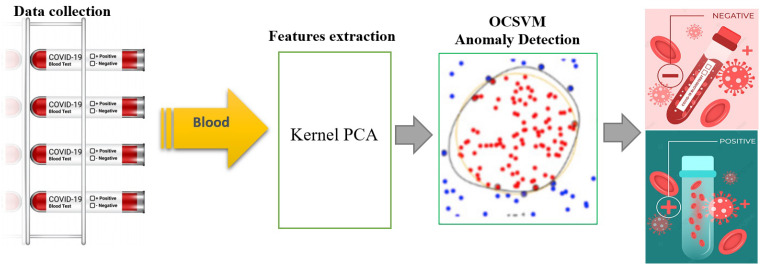
Flowchart of the proposed KPCA-OCSVM anomaly detection scheme.

The proposed KPCA-OCSVM method for identifying COVID-19 infections from blood test data involves training the model using only non-infected samples to extract features. This is done through the use of the KPCA algorithm, which is designed to learn and accurately describe non-infected blood test data and produce relevant features to aid in the training process of the OCSVM algorithm. The OCSVM is then employed to detect infected cases in the testing dataset, which includes both infected and non-infected observations. The features extracted by the KPCA are used as input for the OCSVM, which is sensitive to outliers in the training set (Figure 1). The OCSVM’s objective function is responsible for determining the presence of a COVID-19 infection. The training phase of this approach has a time complexity of O($n^{3}$), and the testing phase has a time complexity of O($n^{2}$), where *n* is the number of samples.

The following seven statistical scores are used in this study to evaluate and compare the performance of the different techniques: true positive rate (TPR), false positive rate (FPR), recall, precision, F1-score, accuracy, and the area under the receiver operating characteristic (ROC) curve (AUC). These scores are calculated using the number of true positives (TPs), false positives (FPs), false negatives (FNs), and true negatives (TNs) obtained from the binary detection results.

### 2.4. Benchmark Methods

In this study, we compared the performance of the introduced KPCA-OCSVM technique against other dimensionality reduction techniques combined with different anomaly detection methods. For the dimensionality reduction part, we considered PCA and ICA, while for the anomaly detection part, we used three commonly used semi-supervised methods, namely EE, LOF, and iForest. These methods were chosen as they have been widely used in various applications and have shown good performance in detecting anomalies. We provide a brief description of each of these techniques in this section.

#### Dimensionality Reduction Methods

**PCA model:** PCA is a linear dimensionality reduction technique that seeks to find the linear combinations of the input variables that explain the most variance in the data [28]. PCA identifies the directions in the data that contain the most information and projects the data onto these directions to reduce the dimensionality [29]. PCA has a time complexity of O($n^{3}$) for both the training and testing phase. This is because PCA relies on the computation of the covariance matrix and the eigenvalues/eigenvectors of that matrix, which both have a time complexity of O($n^{3}$). Specifically, for the training phase, PCA needs to calculate the covariance matrix of the input data, which is of size n∗n, then it needs to compute the eigenvectors and eigenvalues of the covariance matrix, these two steps have O($n^{3}$) time complexity. For the testing phase, once the PCA model is trained, projecting new data points onto the principal components requires matrix multiplication, which also has a time complexity of O($n^{3}$).

**ICA model:** ICA is also a linear method, but it aims to find a linear combination of the original variables, such that the resulting components are statistically independent [30]. ICA is often used to perform blind source separation, which is the task of separating a multivariate signal into independent non-Gaussian components. Essentially, ICA uses kurtosis, which measures the peakedness of a distribution, to find non-Gaussian and independent components [31]. This is because ICA is based on the assumption that the underlying sources of the data are non-Gaussian and independent, and kurtosis is a robust measure of non-Gaussianity. The time complexity of ICA depends on the algorithm used to perform the ICA. Popular algorithms, such as FastICA, have a time complexity of $O(n^{2}p)$, where *n* is the number of samples and *p* is the number of features. Other algorithms such as Infomax have a time complexity of O(nplogn). However, it is worth noting that the time complexity of ICA can also depend on the implementation and specific details of the dataset, such as the number of independent components being estimated. Table 1 compares the main features of the three investigated dimensionality reduction methods, KPCA, PCA, and ICA.

### 2.5. Semi-Supervised Anomaly Detection Methods

**Elliptical envelope (EE):** EE is a density-based anomaly detection algorithm that assumes that the data are generated from a Gaussian distribution. The algorithm fits an ellipse to the data, and any point outside of this ellipse is considered an anomaly [32]. This algorithm is sensitive to the shape of the data distribution and is not suitable for data that do not have Gaussian distributions [33]. The time complexity of EE is $O(n^{3})$ for both the training and testing phase.

**Local outlier factor (LOF):** LOF is a density-based anomaly detection algorithm that calculates the local density of a point compared to its surrounding points [34]. It considers a point an anomaly if its local density is significantly lower than the density of its surrounding points [35]. The time complexity of LOF is $O(n^{2})$ for both the training and testing phase.

**Isolation forest (iForest):** iForest is a tree-based anomaly detection algorithm. It builds a forest of isolation trees, where each tree splits the data based on a randomly selected feature and a random split value [36]. The goal is to isolate anomalies by creating shorter paths for abnormal points, and longer paths for normal points [37]. The iForest algorithm has a computational cost of O(tllogl) during the training phase and O(ntllogl) during the testing phase, where *l* is the subsampling size of the dataset, *n* is the number of samples in the dataset, and *t* is the number of trees in the forest, as reported in [38]. It is worth noting that for optimal detection performance, *l* should be kept small and consistent across different datasets. Table 2 lists the main advantages and shortcomings of the four investigated anomaly detection methods: OCSVM, LOF, iForest, and EE.

## 3. Results and Discussion

### 3.1. Description of the Used Data

This study evaluates the performance of the proposed semi-supervised data-based models for COVID-19 detection using two datasets of blood test samples.

#### 3.1.1. Dataset 1

**Figure 2:**
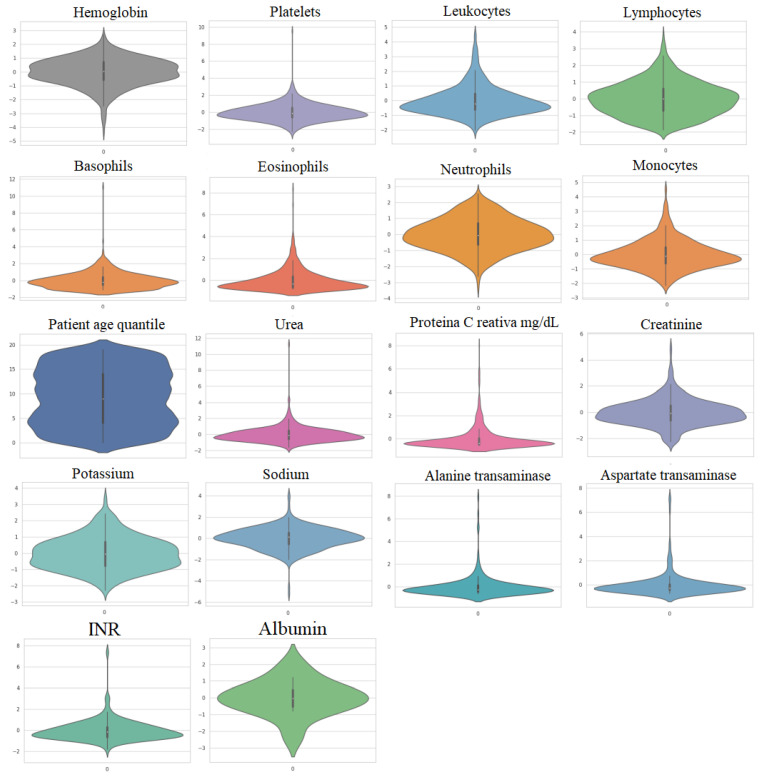
Violin plots of the utilized features in Dataset 1.

The first dataset, referred to as Dataset 1, was gathered from 5644 patients at the Albert Einstein Hospital in São Paulo, Brazil, with 559 of them being COVID-19-positive patients, according to [39]. More details about this dataset can be found in [16,39,40]. Figure 2 illustrates the violin plots of the important 18 features and it can be observed that these datasets do not follow a Gaussian distribution.

**Figure 3:**
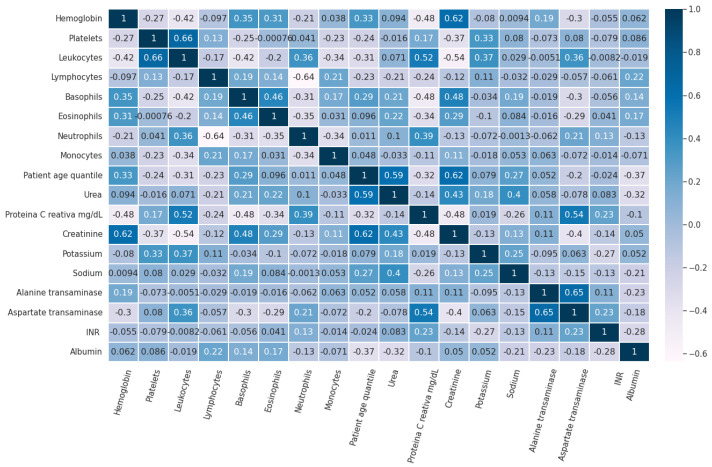
Pairwise Pearson correlation of the features in Dataset 1.

Figure 3 shows the pairwise Pearson correlation of the features in Dataset 1 used for this study.

#### 3.1.2. Dataset 2

**Figure 4:**
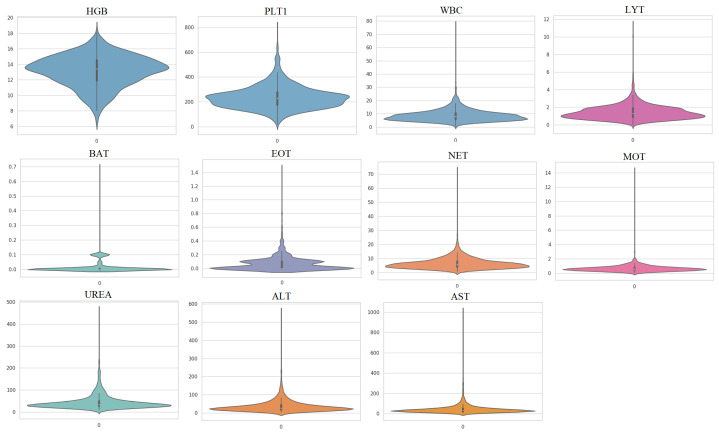
Violin plots of the utilized features in Dataset 2.

In this study, a second dataset was used to evaluate the performance of the proposed methods. This dataset, referred to as Dataset 2, was collected from three different sources and includes hematochemical values from 1624 patients at San Raphael Hospital (OSR) collected between February and May 2020, 58 cases from the Istituto Ortopedico Galeazzi (IOG) of Milan, and 54 patients from OSR in November 2018 [41]. Among the 1624 patients from OSR, 786 were infected with COVID-19 and 838 were uninfected, while 29 of the 58 cases from IOG were infected and 29 were uninfected. The third sub-dataset contains 54 patients from OSR who were not infected with COVID-19 but were used as confounding cases. The use of different instruments to collect samples and the presence of confounding cases make Dataset 2 more challenging compared to Dataset 1. In this study, 11 important features were used to detect COVID-19 infection (Table 3). Figure 4 shows the violin plots of features in Dataset 2 and indicates that these datasets are not Gaussian distributed.

The distribution of the blood test data, as shown in Figure 4, is non-Gaussian, which poses a challenge for traditional dimensionality reduction techniques, such as PCA, which assume linearity and Gaussianity in the data. In this scenario, nonlinear techniques, such as KPCA, which do not have any assumptions on the data distribution, could be more effective.

**Figure 5:**
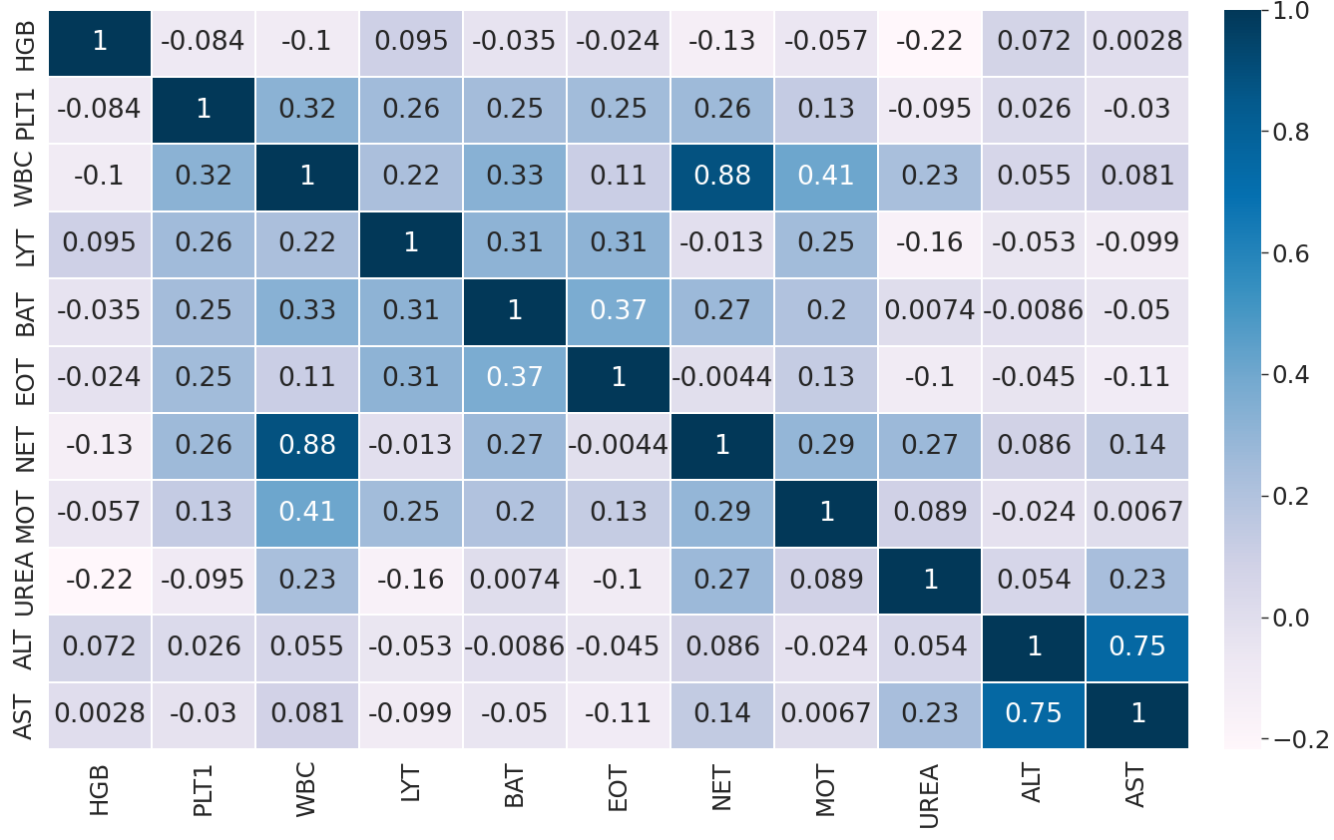
Pairwise Pearson correlation of the features in Dataset 2.

The pairwise Pearson correlation matrix of the features in Dataset 1 is displayed in Figure 5.

### 3.2. Detection Results

The detection performance of various methods, including KPCA-OCSVM, PCA-OCSVM, ICA-OCSVM, iForest, LOF, and EE, were evaluated and compared using Table 4. In this study, four variants of the OCSVM are investigated. The four kernels used in these variants are RBF, polynomial, sigmoid, and linear. They are denoted as OCSVMRBF, OCSVMPoly, OCSVMSig, and OCSVMLin, respectively. These detectors were trained in a semi-supervised manner, using only non-infected blood test data for training. The training set consisted of 85% of non-infected observations, while the test set included the remaining 15% of non-infected observations and all infected observations. In the training phase of PCA and ICA, it was determined that using three principal components resulted in satisfactory performance. Here is the setting of KPCA (number of components = 5, kernel = ‘RBF’, gamma = 0.1). We used the grid search approach to determine the optimal values of these parameters. In the training phase of the four anomaly detection techniques, we used the following parameter settings: isolation forest (number of estimators = 150, contamination rate = 0.05), OCSVM (kernel = RBF, nu = 0.001, gamma = 0.05), local outlier factor (novelty detection = true, number of neighbors = 20, metric = ‘Minkowski’, contamination = 0.1), and elliptical envelope (support fraction = 0.25). All experiments were conducted on a laptop with an i3 processor running Ubuntu 20.04.4 LTS, with 8GB of RAM, to ensure a fair comparison. The methods were implemented using Python 3.8, and the Keras and scikit-learn libraries, version 0.22.

As per Table 4, the KPCA-OCSVMRBF, PCA-OCSVMRBF, and ICA-OCSVMRBF methods showed excellent detection performance with an AUC of 0.99. They were followed by the ICA-based EE method with an AUC of 0.82. The other methods did not provide satisfactory results. In terms of all metrics, the KPCA-OCSVMRBF, PCA-OCSVMRBF, and ICA-OCSVMRBF detectors performed the best in detecting COVID-19 infection based on Dataset 1. The results showed that combining PCA, ICA, and KCA with OCSVM${}_{\mathrm{RBF}}$ provided better performance than the other combinations of dimensionality reduction techniques and OCSVM kernels. This indicates that using OCSVM with an RBF kernel with PCA, ICA, and KCA can effectively capture the non-linear relationships between the data points, resulting in improved anomaly detection performance. The results also suggest that using dimensionality reduction techniques as feature extractors followed by the OCSVMRBF algorithm provides better detection accuracy than other semi-supervised detectors, such as iForest, LOF, and EE. It is worth mentioning that this research focuses on identifying COVID-19 infections using blood test results as an outlier detection task, and the semi-supervised method proposed has shown to be highly effective in detecting contaminated cases.

One possible explanation for the superior performances of KPCA-OCSVM, PCA-OCSVM, and ICA-OCSVM methods is that they combine the advantages of both dimensionality reduction techniques and the OCSVM algorithm. The use of dimensionality reduction models as feature extractors allows these methods to reduce the dimensionality of the data, which can help to improve the detection performance by reducing noise and irrelevant information. Additionally, the OCSVM algorithm used in these methods can detect abnormal features in the data, which further improves the detection performance. Importantly, the use of OCSVM as an anomaly detector performs better than other semi-supervised detectors, such as iForest, LOF, and EE. This could be due to the flexibility of OCSVM to map the data to higher-dimensional spaces via non-linear kernels, making it easier to separate the normal data from anomalies. EE assumes that data will follow a multivariate Gaussian distribution, while OCSVM, iForest, and LOF are non-parametric and, therefore, more robust to deviations from the assumed distribution. However, iForest and LOF use distance-based approaches and may struggle with non-linear relationships. The use of semi-supervised learning in these methods allows them to leverage information from non-infected observations to improve the detection performance.

To enhance the evaluation of the proposed KPCA-OCSM approach’s detection performance, we utilized the bootstrap method to calculate confidence intervals for performance metrics. This involved generating “nboots” bootstrap sample datasets; each was the same size as the original test set. To create these sample datasets, instances were randomly drawn from the test set with replacements. Evaluation metrics were calculated for each sample dataset, and the 95% confidence interval was determined by computing the 2.5th to the 97.5th percentile among the “nboots” calculated metric values.

**Figure 6:**
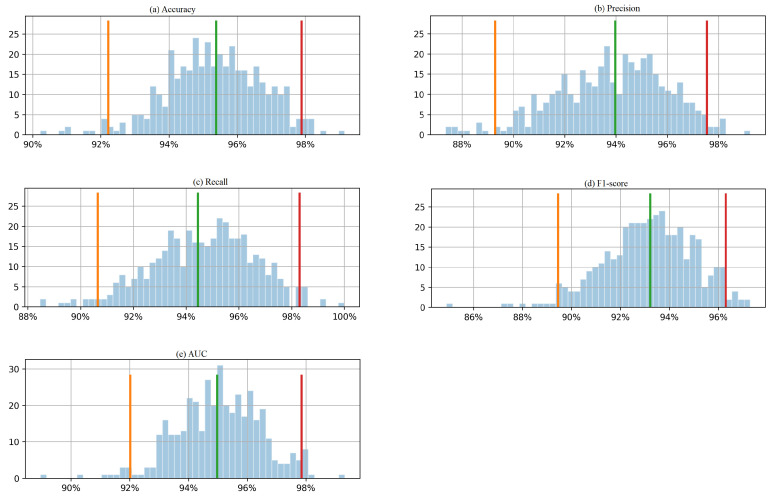
Histogram of 400 bootstrapped outcomes with a 95% confidence interval for each evaluation metric (**a**) accuracy, (**b**) precision, (**c**) recall, (**d**) F1-score, and (**e**) AUC, calculated from the testing Dataset 1.

In this study, we utilized the bootstrap method with 400 boots to calculate the 95% confidence intervals for 5 evaluation metrics based on the test data. Figure 6 presents the histogram of the 400 boot results, depicting a 95% confidence interval. The histogram shows the frequency distribution of the bootstrap results. The orange and red lines on the histogram represent the lower and upper bounds of the 95% confidence interval, respectively. The confidence interval is a range of values that is likely to contain the true value of the population parameter with a certain level of confidence. For example, the 95% confidence interval of accuracy and AUC is between around 92% and 98%, which means that the true values of accuracy and AUC for the population are likely to fall within this range with 95% confidence. Similarly, the precision is between around 89% and 98%, which means that the true value of precision for the population is likely to fall within this range with 95% confidence. The same applies to recall and F1-score. From Table 5, the accuracy, precision, recall, F1-score, and AUC values obtained from the real testing data are 1, 1, 1, 1, and 0.99, respectively, means that the model’s performance on the test data is excellent. However, it is important to note that the confidence intervals calculated using the bootstrap method are based on a range of values that the evaluation metrics could take in repeated sampling from the same population. The confidence intervals give an estimate of the precision of the estimates, taking into account the variability in the sample data. In this case, the 95% confidence interval of accuracy and AUC was between 92% and 98%, while the precision was between 89% and 98%. The perfect scores obtained from the real testing data fall outside the range of these confidence intervals. This could be because the sizes of the testing data were relatively small.

In the second experiment, we evaluated the effectiveness of the KPCA-OCSVM model in detecting COVID-19 infection using Dataset 2. The results, as shown in Table 5, indicate that the KPCA-OCSVM detector performed well with an AUC of 0.99. Compared to other KPCA-based models, such as iForest, LOF, and EE detectors, the KPCA-OCSVM model achieved superior results. Additionally, when comparing KPCA-OCSVM, PCA-OCSVM, ICA-OCSVM, iForest, LOF, and EE detectors, the KPCA-OCSVM and PCA-OCSVM models provided the highest detection performance with an AUC of 0.99, followed by the ICA-OCSVM detector with an AUC of 0.97. These results demonstrate the potential of the KPCA-based OCSVM model for detecting COVID-19 infection using blood test data.

**Figure 7:**
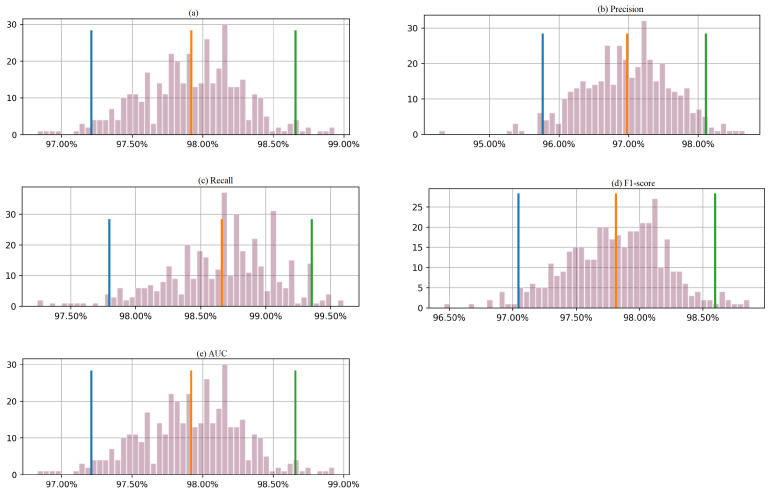
Histogram of 400 bootstrapped outcomes with a 95% confidence interval for each evaluation metric (**a**) accuracy, (**b**) precision, (**c**) recall, (**d**) F1-score, and (**e**) AUC, calculated from the testing Dataset 2.

Figure 7 presents the results based on a bootstrapping technique that produced 400 outcomes, with a 95% confidence interval for each metric. The orange and red lines on the histogram represent the lower and upper bounds of the 95% confidence interval, respectively. For accuracy, the 95% confidence interval ranges from 97.2% to 98.7%. Similarly, the confidence interval for precision is between 95.8% and 98.1%. The 95% confidence interval for the recall is between 97.75% and 99.4%, while that for F1-score is approximately between 97.1% and 98.6%. For AUC, the confidence interval is between 97.20% and 98.6%. In this case, the actual performance of the proposed approach on the test data is slightly outside the calculated confidence intervals. One possible explanation for the model’s excellent performance on the test data relative to the bootstrapped samples could be that the test data are more representative samples of the populations than the bootstrapped samples. In that case, this may indicate that the model is better suited to the characteristics of the test data. This could be due to a variety of reasons, such as differences in the distribution of the features or the target variable or differences in the data collection process. Another possible explanation is that the dataset is small. In this case, the model’s performance may be more variable, and the calculated confidence intervals may be wider. This means that the model’s actual performance may deviate from the expected performance based on the bootstrapped samples.

### 3.3. Comparison with the Existing Methods

In this study, the performance of the developed KPCA-OCSVM detector was evaluated against a variety of current techniques applied to both Dataset 1 and Dataset 2, as detailed in Table 6. Previous studies, such as the one in [40], employed supervised machine learning techniques, including RF, ANN, logistic regression, and lasso-elastic-net regularized generalized linear (GLMNET) models, to predict SARS-CoV-2 infection. While ANN achieved the best classification results with an AUC of 0.95, other studies, such as [19,42], also applied various supervised machine learning techniques, such as NN, RF, GBT, SVM, MLP, RT, BN, and Naive Bayes. Despite the high overall accuracy of 95.159% achieved by BN, these methods all require labeled data for classification. In contrast, the proposed KPCA-OCSVM detector is a semi-supervised method, which utilizes unlabeled data during the training process, making it a suitable solution for detecting COVID-19 infections. According to Table 6, the KPCA-OCSVM method exhibits superior performance in comparison to the state-of-the-art methods, displaying a noteworthy ability to detect infection in both datasets.

In summary, the proposed KPCA-OCSVM method offers several benefits, making it a promising approach for detecting COVID-19 infection using blood test data. Firstly, it is a semi-supervised approach, which only requires data from healthy cases during training. This makes it easier to implement and less time-consuming than supervised methods that require labeled data, which can be challenging to obtain in practice. Moreover, labeling data can be subjective and prone to errors, making semi-supervised methods a more robust alternative. Secondly, the KPCA-OCSVM method can detect unseen anomalies, which is a key advantage in the context of detecting COVID-19 infections. Since the COVID-19 virus constantly evolves, the KPCA-OCSVM method can identify new strains and variants that were absent during the training phase. This is in contrast to supervised methods that are limited to detecting only known anomalies. Thirdly, the KPCA-OCSVM method is able to identify nonlinear patterns in the data. This is important in the case of blood test data, which may be non-Gaussian distributed and contain complex relationships between the features. By using KPCA, the method can identify these nonlinear patterns and capture the underlying structure of the data more accurately. Lastly, the results of this study reveal that the KPCA-OCSVM method outperforms the state-of-the-art methods in terms of detection performance, as shown in Table 6. This indicates that the proposed approach is more accurate and reliable in detecting COVID-19 infections using blood test data.

### 3.4. Feature Importance Identification

To understand the importance of different variables in COVID-19 infection, we used XGBoost (extreme gradient boosting) and (SHapley Additive exPlanations) values to analyze the importance of blood sample variables for COVID-19 contamination detection. This approach allows researchers to analyze a large amount of data and determine which variables are most strongly associated with COVID-19 infection, providing valuable insights into the underlying mechanisms of the disease.

XGBoost is a popular machine learning algorithm that can handle complex, non-linear relationships in data, making it well-suited for analyzing the complex relationships between blood sample variables and COVID-19 infection. Specifically, XGBoost builds a series of decision trees, each trained to predict the outcome variable. The final prediction is obtained by combining the predictions of all the trees. This allows XGBoost to capture complex relationships between the input and outcome variables, making it well-suited for analyzing the complex relationships between blood sample variables and COVID-19 infection. SHAP values, on the other hand, are model-agnostic techniques for interpreting the output of any machine learning model, including XGBoost. Importantly, SHAP values measure each variable’s contribution to the predicted outcome, allowing researchers to identify which variables are most important for predicting COVID-19 infection. We can identify which variables are most strongly associated with COVID-19 infection by calculating each variable’s mean absolute SHAP values. Variables with high SHAP values are more important for predicting COVID-19 infection, and understanding the mechanisms behind these variables can provide valuable insights into the disease. Essentially, the importance of identifying the most important variables for COVID-19 infection detection lies in the ability to develop more accurate diagnostic tools. By combining the most important variables, researchers can develop diagnostic tests that are more accurate and efficient in detecting COVID-19 infection.

**Figure 8:**
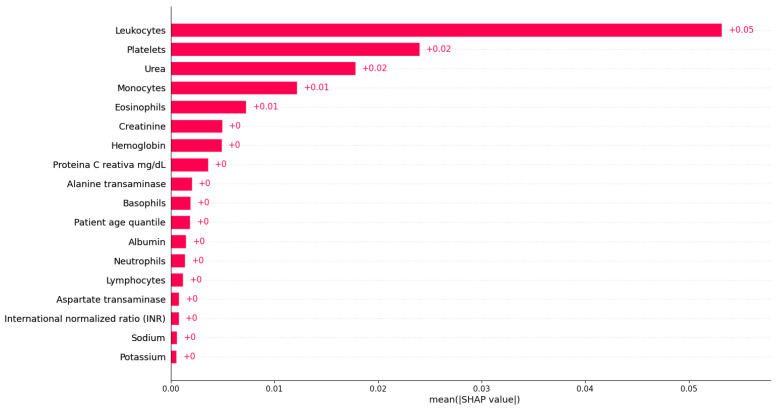
Feature importance identification using XGBoost based on Dataset 1.

Figure 8 shows the bar plot of the mean absolute SHAP values for each variable based on Dataset 1. From Figure 8, we can observe that the most important feature for detecting COVID-19 infection in Dataset 1 is ‘Leukocytes’. Indeed, ‘Leukocytes’ are white blood cells that play a crucial role in the body’s immune system response, so it is not unexpected that they are essential in predicting COVID-19. ‘Platelets’ is identified as the second most important variable in detecting COVID-19 infection, as shown in Figure 8. Platelets are blood cells that are crucial in clotting and wound healing. The significance of these variables in predicting COVID-19 is not surprising, as leukocytes are essential in the body’s immune response, and platelets are necessary for clotting and wound healing, both of which play a crucial role in combating viral infections [1]. The next identified most important features are ‘Urea’, ‘Monocytes’, and ‘Eosinophils’, respectively. ‘Urea’ is a waste product filtered by the kidneys, and abnormal levels can indicate kidney dysfunction or dehydration. Additionally, ‘Monocytes’ and ‘Eosinophils’ are types of white blood cells that are involved in the body’s immune response to viral infections, indicating the importance of the immune system in COVID-19. Monocytes are white blood cells that play an important role in the immune system’s response to infections, including viral infections such as COVID-19 [15]. The identification result suggests that changes in monocyte levels or functions may be significant in determining whether a person is infected with the virus. On the other hand, eosinophils play a role in the body’s response to parasitic infections, allergic reactions, and inflammation [43]. In COVID-19, the virus can trigger an overactive immune response called a cytokine storm, leading to inflammation and tissue damage, especially in the lungs [15]. Although ‘Creatinine’, ‘Hemoglobin’, and ‘proteina C reativa’ have relatively low importance in detecting COVID-19 infection compared to the other variables, they still may play a role in the disease and warrant further investigation. ‘Creatinine’ is another waste product that is filtered by the kidneys and can indicate kidney dysfunction or dehydration. ‘Hemoglobin’ is a protein found in red blood cells that carries oxygen throughout the body, and low levels can indicate anemia, which may be a complication of severe COVID-19. ‘Proteina C reativa’ is a marker of inflammation in the body and can be elevated in COVID-19 patients. The remaining features with low importance may still be useful in predicting COVID-19; they may not be as strongly associated with the disease as the other features listed above. Further research into the relationship between these variables and COVID-19 can provide additional insights into the mechanisms of the disease and potentially guide treatment decisions. Overall, by analyzing the most important variables for predicting COVID-19 infection using XGBoost and SHAP values, we could obtain a better understanding of how the virus affects the body and can identify potential targets for treatment.

**Figure 9:**
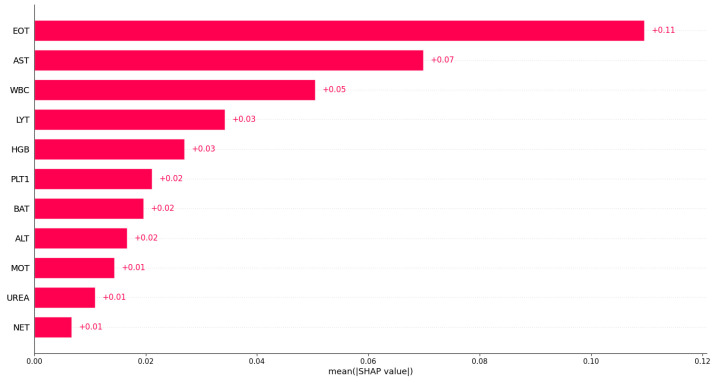
Feature importance identification using XGBoost based on Dataset 2.

Similarly, we investigated feature importance based on Dataset 2; Figure 9 displays the bar plot of the mean absolute SHAP values. Based on Dataset 2, the eosinophil count was identified as the most important variable in predicting COVID-19. This indicates that the eosinophil count may play a significant role in distinguishing between COVID-19 patients and non-COVID-19 patients in that dataset. From Figure 9, we can see that aspartate aminotransferase (AST), white blood cells (WBC), and lymphocyte count (LYT) were also identified as important variables in predicting COVID-19. This suggests that these variables may also play a role in discriminating between COVID-19 patients and non-COVID-19 patients in that dataset. However, the exact impact of these variables on predicting COVID-19 may vary in different datasets or models, and additional research may be needed to fully understand their relationships with COVID-19. A full analysis of the importance of blood sample variables in detecting COVID-19 infection is beyond the scope of the current paper.

The feature importance identification results align with the existing literature on COVID-19. Studies have reported that COVID-19 patients often exhibit lymphopenia, liver and muscle damage, and elevated C-reactive protein (CRP) levels [44,45,46]. Additionally, various studies have reported common abnormalities in COVID-19 patients, such as increased AST, decreased lymphocyte count, increased white blood cell count (WBC), and increased ALT [18,47].

Identifying feature importance using the XGBoost algorithm can be a helpful tool in predicting COVID-19 infection from blood test data. The algorithm can identify which features are most strongly associated with COVID-19 infection by analyzing many features simultaneously. This information can be used to develop more accurate diagnostic tools for COVID-19 and to identify new biomarkers that may be useful in predicting infection or monitoring disease progression. Additionally, identifying feature importance can help researchers better understand the underlying biological mechanisms of COVID-19 infection. By identifying which features are most important in predicting infection, researchers can gain insights into the specific pathways and processes affected by the virus. This information can be used to develop new treatments or therapies that target these specific pathways, potentially improving patient outcomes. For example, if the algorithm identifies white blood cell counts as the most important feature, this may indicate that the immune response is a key factor in the disease. Researchers can then use this information to investigate the specific mechanisms by which the virus affects the immune system, potentially leading to the development of new treatments or therapies. In summary, by providing insights into the underlying biological mechanisms of the disease and identifying new biomarkers for diagnosis and treatment, this approach can help healthcare professionals and researchers better understand, diagnose, and treat COVID-19.

While XGBoost and SHAP values can provide valuable insights into the importance of different variables in COVID-19 infection, it is important to further investigate and validate the results with the help of medical experts. The most important variable identified by the algorithm should be carefully examined to determine whether it makes physiological sense and whether it aligns with existing knowledge about the disease. It is also crucial to consider potential confounding variables that may impact the results and to carefully design experiments to validate the findings. Overall, using XGBoost and SHAP values to identify the most important variables for COVID-19 detection is just the first step in a rigorous process that requires collaboration between data scientists and medical experts to ultimately develop effective diagnostic tests and treatment strategies.

This study highlights that semi-supervised machine learning methods can be essential in detecting COVID-19 from blood test data. Machine learning methods can analyze large amounts of data quickly and accurately, providing valuable insights that can aid in diagnosing COVID-19. By analyzing blood test data, machine learning models can identify specific biomarkers indicative of COVID-19 infection. These biomarkers can include white blood cell counts, levels of specific enzymes, and other indicators of inflammation or organ dysfunction. By using machine learning to analyze these biomarkers, healthcare professionals can quickly and accurately identify patients who may be infected with COVID-19, allowing for earlier intervention and treatment. Additionally, machine learning algorithms can continually learn from new data, improving accuracy and providing more effective diagnostic tools for COVID-19 detection. Overall, the use of semi-supervised machine learning methods for COVID-19 detection in blood test data have the potential to improve patient outcomes and aid in global efforts to control the spread of the disease.

## 4. Conclusions

This study proposes a novel method for detecting COVID-19 infection using blood test data as part of an anomaly detection problem. The KPCA-OCSVM approach combines KPCA and semi-supervised OCSVM techniques to detect infected cases without requiring labeled data. The KPCA method identifies nonlinear patterns, while the OCSVM measures dissimilarity between normal and abnormal features. The approach outperformed other semi-supervised models when tested on two datasets of blood test samples from hospitals in Brazil and Italy. The proposed method shows promise for detecting COVID-19 infection using blood test data without labeled data.

While the proposed approach for detecting COVID-19 infections based on blood test data shows promise, some limitations need to be addressed. One of the main limitations is that the study only tested the approach on two sets of blood test samples from hospitals in Brazil and Italy. Therefore, it is necessary to validate the approach on more diverse datasets from different regions and populations to ensure its generalizability and reliability. Moreover, incorporating deep learning techniques, such as deep generative models, could potentially improve the ability of the method to detect anomalies in the data. This could include using generative adversarial networks (GANs) [48] to learn more complex representations of the data. These deep learning techniques have been shown to be effective for anomaly detection in other domains and could be useful for detecting COVID-19 infections based on blood test data. Additionally, incorporating other relevant features, such as demographic information and medical history, could also be explored to improve the accuracy of the model. These features could provide additional insights into the factors that contribute to COVID-19 infections and help to develop a more robust and accurate detection model. Finally, as new virus variants emerge, it will be necessary to continually update and validate detection methods based on blood test data to ensure their effectiveness. This highlights the importance of ongoing research and development in this area to keep up with the evolving nature of the COVID-19 pandemic.

## Figures and Tables

**Table 1:** Comparison between KPCA, PCA, and ICA.

Method	Time Complexity	Linear/Nonlinear	Data Distribution
KPCA	O($n^{3}$) or O($n^{2}$)	Nonlinear	Non-Gaussian
PCA	O($n^{3}$) or O($n^{2}$)	Linear	Gaussian
ICA	O($n^{3}$)	Linear	Non-Gaussian

**Table 2:** Summary of advantages and shortcomings of four anomaly detection schemes.

Approach	Advantages	Shortcomings
OCSVM	- Assumption-free about the data distribution	- Sensitive to the choice of the kernel function and parameters
iForest	- High-dimensional data and large datasets are supported	- Prone to overfitting and may not handle circular patterns well
LOF	- Can handle multi-dimensional data	- Sensitive to the choice of parameters and may not handle noisy data well
EE	- Assumes normal data are distributed normally	- Sensitive to the choice of parameters and assumes data are distributed normally

**Table 3:** The used features in Dataset 2.

Feature	Abbreviation
Hemoglobin	HGB
Platelets	PLT1
White blood cells	WBC
Lymphocyte count	LYT
Basophils count	BAT
Eosinophil count	EOT
Neutrophil count	NET
Monocyte count	MOT
Urea	Urea
Alanine aminotransferase	ALT
Aspartate aminotransferase	AST

**Table 4:** Detection results based on testing data in Dataset 1.

Approach	TPR	FPR	Accuracy	Precision	Recall	F1Score	AUC
KPCA-OCSVM${}_{\mathrm{RBF}}$	1.00	0.03	1.00	1.00	1.00	1.00	0.99
KPCA-OCSVM${}_{\mathrm{Poly}}$	0.37	0.40	0.51	0.35	0.37	0.36	0.48
KPCA-OCSVM${}_{\mathrm{Sig}}$	0.34	0.44	0.45	0.43	0.34	0.38	0.45
KPCA-OCSVM${}_{\mathrm{Lin}}$	0.46	0.35	0.58	0.44	0.46	0.45	0.56
KPCA-iForest	0.93	0.83	0.68	0.70	0.93	0.80	0.55
KPCA-LOF	0.95	0.45	0.91	0.95	0.95	0.95	0.75
KPCA-EE	1.00	0.47	0.91	0.90	1.00	0.95	0.76
PCA-OCSVM${}_{\mathrm{RBF}}$	1.00	0.03	1.00	1.00	1.00	1.00	0.99
PCA-OCSVM${}_{\mathrm{Poly}}$	0.39	0.39	0.52	0.41	0.39	0.40	0.50
PCA-OCSVM${}_{\mathrm{Sig}}$	0.33	0.45	0.44	0.43	0.33	0.37	0.44
PCA-OCSVM${}_{\mathrm{Lin}}$	0.20	0.61	0.29	0.28	0.20	0.23	0.29
PCA-iForest	0.91	0.87	0.66	0.69	0.91	0.79	0.52
PCA-LOF	0.94	0.52	0.90	0.94	0.94	0.94	0.71
PCA-EE	1.00	0.48	0.91	0.90	1.00	0.95	0.76
ICA-OCSVM${}_{\mathrm{RBF}}$	1.00	0.03	1.00	1.00	1.00	1.00	0.99
ICA-OCSVM${}_{\mathrm{Poly}}$	0.39	0.39	0.51	0.43	0.39	0.41	0.50
ICA-OCSVM${}_{\mathrm{Sig}}$	0.39	0.39	0.48	0.56	0.39	0.46	0.50
ICA-OCSVM${}_{\mathrm{Lin}}$	0.14	0.80	0.16	0.22	0.14	0.17	0.17
ICA-ISOL	0.91	0.87	0.73	0.78	0.91	0.84	0.52
ICA-LOF	0.94	0.48	0.90	0.95	0.94	0.95	0.73
ICA-EE	1.00	0.36	0.94	0.94	1.00	0.97	0.82

**Table 5:** Summary of advantages and shortcomings of four anomaly detection schemes.

Approach	TPR	FPR	Accuracy	Precision	Recall	F1Score	AUC
KPCA-OCSVM${}_{\mathrm{RBF}}$	0.97	0.00	0.99	1.00	0.97	0.99	0.99
KPCA-OCSVM${}_{\mathrm{Poly}}$	0.71	0.98	0.23	0.24	0.71	0.36	0.36
KPCA-OCSVM${}_{\mathrm{Sig}}$	0.90	0.90	0.51	0.51	0.90	0.65	0.50
KPCA-OCSVM${}_{\mathrm{Lin}}$	0.77	0.96	0.29	0.29	0.77	0.43	0.41
KPCA-iForest	0.55	0.23	0.66	0.71	0.55	0.62	0.66
KPCA-LOF	0.81	0.10	0.86	0.85	0.81	0.83	0.85
KPCA-EE	0.73	0.10	0.82	0.87	0.73	0.79	0.82
PCA-OCSVM${}_{\mathrm{RBF}}$	0.97	0.00	0.99	1.00	0.97	0.99	0.99
PCA-OCSVM${}_{\mathrm{Poly}}$	0.73	0.96	0.23	0.22	0.73	0.34	0.38
PCA-OCSVM${}_{\mathrm{Sig}}$	0.89	0.91	0.50	0.50	0.89	0.64	0.49
PCA-OCSVM${}_{\mathrm{Lin}}$	0.62	0.99	0.16	0.16	0.62	0.26	0.32
PCA-iForest	0.60	0.20	0.70	0.74	0.60	0.66	0.70
PCA-LOF	0.78	0.10	0.85	0.85	0.78	0.82	0.84
PCA-EE	0.71	0.10	0.81	0.87	0.71	0.78	0.81
ICA-OCSVM${}_{\mathrm{RBF}}$	0.94	0.01	0.97	0.99	0.94	0.96	0.97
ICA-OCSVM${}_{\mathrm{Poly}}$	0.70	0.98	0.22	0.22	0.70	0.34	0.36
ICA-OCSVM${}_{\mathrm{Sig}}$	0.87	0.93	0.49	0.51	0.87	0.64	0.47
ICA-OCSVM${}_{\mathrm{Lin}}$	0.96	0.71	0.79	0.79	0.96	0.87	0.62
ICA-ISOL	0.60	0.20	0.71	0.72	0.60	0.66	0.70
ICA-LOF	0.75	0.09	0.84	0.88	0.75	0.81	0.83
ICA-EE	0.71	0.10	0.81	0.87	0.71	0.78	0.81

**Table 6:** Comparison with existing methods.

Refs	Dataset	Model	Metrics
[40]	Dataset 1	RF, LR, GLMNET, and ANN	AUC = 95
[42]	Dataset 1	NN, RF, GBT, LR, and SVM	AUC = 85
[19]	Dataset 1	MLP, SVM, RT, RF, BN, and NB	Acc = 95.15%
			Sens = 96.8%, Spec = 93.6%
[16]	Dataset 1	XGBoost	AUC = 99.38
**KPCA-OCSVM**	Dataset 1	**KPCA-OCSVM**	**AUC = 99**
[41]	Dataset 2	DT-XGBoost	AUC = 85
**KPCA-OCSVM**	Dataset 2	**KPCA-OCSVM**	**AUC = 99**

## Data Availability

Not applicable.

## References

[1] Kistenev Y.V., Vrazhnov D.A., Shnaider E.E., Zuhayri H. (2022). Predictive models for COVID-19 detection using routine blood tests and machine learning. Heliyon.

[2] Day M. (2020). COVID-19: Identifying and isolating asymptomatic people helped eliminate virus in Italian village. BMJ Br. Med. J..

[3] Rikan S.B., Azar A.S., Ghafari A., Mohasefi J.B., Pirnejad H. (2022). COVID-19 diagnosis from routine blood tests using artificial intelligence techniques. Biomed. Signal Process. Control.

[4] Chadaga K., Prabhu S., Vivekananda Bhat K., Umakanth S., Sampathila N. (2022). Medical diagnosis of COVID-19 using blood tests and machine learning. J. Phys. Conf. Ser..

[5] Lee Y., Kim Y.S., Lee D.i., Jeong S., Kang G.H., Jang Y.S., Kim W., Choi H.Y., Kim J.G., Choi S.h. (2022). The application of a deep learning system developed to reduce the time for RT-PCR in COVID-19 detection. Sci. Rep..

[6] Loddo A., Meloni G., Pes B. (2022). Using Artificial Intelligence for COVID-19 Detection in Blood Exams: A Comparative Analysis. IEEE Access.

[7] Wang B., Zhao Y., Chen C.P. (2021). Hybrid Transfer Learning and Broad Learning System for Wearing Mask Detection in the COVID-19 Era. IEEE Trans. Instrum. Meas..

[8] Sharma R.R., Kumar M., Maheshwari S., Ray K.P. (2020). EVDHM-ARIMA-based time series forecasting model and its application for COVID-19 cases. IEEE Trans. Instrum. Meas..

[9] Lam C., Tso C.F., Green-Saxena A., Pellegrini E., Iqbal Z., Evans D., Hoffman J., Calvert J., Mao Q., Das R. (2021). Semisupervised deep learning techniques for predicting acute respiratory distress syndrome from time-series clinical data: Model development and validation study. JMIR Form. Res..

[10] Wu W., Shi J., Yu H., Wu W., Vardhanabhuti V. (2021). Tensor gradient L_0_-norm minimization-based low-dose CT and its application to COVID-19. IEEE Trans. Instrum. Meas..

[11] Han C.H., Kim M., Kwak J.T. (2021). Semi-supervised learning for an improved diagnosis of COVID-19 in CT images. PLoS ONE.

[12] Khobahi S., Agarwal C., Soltanalian M. (2020). Coronet: A deep network architecture for semi-supervised task-based identification of covid-19 from chest x-ray images. MedRxiv.

[13] Brunese L., Mercaldo F., Reginelli A., Santone A. (2020). Explainable deep learning for pulmonary disease and coronavirus COVID-19 detection from X-rays. Comput. Methods Programs Biomed..

[14] Dairi A., Harrou F., Sun Y. (2021). Deep generative learning-based 1-svm detectors for unsupervised COVID-19 infection detection using blood tests. IEEE Trans. Instrum. Meas..

[15] Alves M.A., Castro G.Z., Oliveira B.A.S., Ferreira L.A., Ramírez J.A., Silva R., Guimar aes F.G. (2021). Explaining machine learning based diagnosis of COVID-19 from routine blood tests with decision trees and criteria graphs. Comput. Biol. Med..

[16] AlJame M., Ahmad I., Imtiaz A., Mohammed A. (2020). Ensemble learning model for diagnosing COVID-19 from routine blood tests. Inform. Med. Unlocked.

[17] Sargiani V., De Souza A.A., De Almeida D.C., Barcelos T.S., Munoz R., Da Silva L.A. (2022). Supporting Clinical COVID-19 Diagnosis with Routine Blood Tests Using Tree-Based Entropy Structured Self-Organizing Maps. Appl. Sci..

[18] Brinati D., Campagner A., Ferrari D., Locatelli M., Banfi G., Cabitza F. (2020). Detection of COVID-19 infection from routine blood exams with machine learning: A feasibility study. J. Med. Syst..

[19] de Freitas Barbosa V.A., Gomes J.C., de Santana M.A., Albuquerque J.E.d.A., de Souza R.G., de Souza R.E., dos Santos W.P. (2022). Heg. IA: An intelligent system to support diagnosis of Covid-19 based on blood tests. Res. Biomed. Eng..

[20] Aktar S., Ahamad M.M., Rashed-Al-Mahfuz M., Azad A., Uddin S., Kamal A., Alyami S.A., Lin P.I., Islam S.M.S., Quinn J.M. (2021). Machine Learning Approach to Predicting COVID-19 Disease Severity Based on Clinical Blood Test Data: Statistical Analysis and Model Development. JMIR Med. Inform..

[21] Choi S.W., Lee C., Lee J.M., Park J.H., Lee I.B. (2005). Fault detection and identification of nonlinear processes based on kernel PCA. Chemom. Intell. Lab. Syst..

[22] Hejazi M., Singh Y.P. (2013). One-class support vector machines approach to anomaly detection. Appl. Artif. Intell..

[23] Harrou F., Sun Y., Hering A.S., Madakyaru M., Dairi A. (2021). Unsupervised deep learning-based process monitoring methods. Statistical Process Monitoring Using Advanced Data-Driven and Deep Learning Approaches.

[24] Schölkopf B., Platt J.C., Shawe-Taylor J., Smola A.J., Williamson R.C. (2001). Estimating the support of a high-dimensional distribution. Neural Comput..

[25] Alam S., Sonbhadra S.K., Agarwal S., Nagabhushan P. (2020). One-class support vector classifiers: A survey. Knowl.-Based Syst..

[26] Sebald D.J., Bucklew J.A. (2000). Support vector machine techniques for nonlinear equalization. IEEE Trans. Signal Process..

[27] Schölkopf B., Smola A., Müller K.R. (2005). Kernel principal component analysis. Artificial Neural Networks—Proceedings of the ICANN’97: 7th International Conference Lausanne, Switzerland, 8–10 October 1997 Proceeedings.

[28] Harrou F., Kadri F., Khadraoui S., Sun Y. (2016). Ozone measurements monitoring using data-based approach. Process. Saf. Environ. Prot..

[29] Harrou F., Nounou M.N., Nounou H.N., Madakyaru M. (2013). Statistical fault detection using PCA-based GLR hypothesis testing. J. Loss Prev. Process. Ind..

[30] Kong X., Yang Z., Luo J., Li H., Yang X. (2022). Extraction of Reduced Fault Subspace Based on KDICA and Its Application in Fault Diagnosis. IEEE Trans. Instrum. Meas..

[31] Hyvärinen A., Oja E. (2000). Independent component analysis: Algorithms and applications. Neural Netw..

[32] Rousseeuw P.J., Driessen K.V. (1999). A fast algorithm for the minimum covariance determinant estimator. Technometrics.

[33] Dairi A., Zerrouki N., Harrou F., Sun Y. (2022). EEG-Based Mental Tasks Recognition via a Deep Learning-Driven Anomaly Detector. Diagnostics.

[34] Breunig M.M., Kriegel H.P., Ng R.T., Sander J. LOF: Identifying density-based local outliers. Proceedings of the 2000 ACM SIGMOD International Conference on Management of Data.

[35] Dairi A., Harrou F., Sun Y. (2022). Efficient Driver Drunk Detection by Sensors: A Manifold Learning-Based Anomaly Detector. IEEE Access.

[36] Liu F.T., Ting K.M., Zhou Z.H. Isolation forest. Proceedings of the 2008 Eighth IEEE International Conference on Data Mining.

[37] Liu F.T., Ting K.M., Zhou Z.H. (2012). Isolation-based anomaly detection. ACM Trans. Knowl. Discov. Data.

[38] Chabchoub Y., Togbe M.U., Boly A., Chiky R. (2022). An in-depth study and improvement of Isolation Forest. IEEE Access.

[39] Data4u E. Diagnosis of COVID-19 and Its Clinical Spectrum AI and Data Science Supporting Clinical Decisions (from 28 March to 3 April). https://www.kaggle.com/einsteindata4u/covid19.

[40] Banerjee A., Ray S., Vorselaars B., Kitson J., Mamalakis M., Weeks S., Baker M., Mackenzie L.S. (2020). Use of machine learning and artificial intelligence to predict SARS-CoV-2 infection from full blood counts in a population. Int. Immunopharmacol..

[41] Cabitza F., Campagner A., Ferrari D., Di Resta C., Ceriotti D., Sabetta E., Colombini A., De Vecchi E., Banfi G., Locatelli M. (2021). Development, evaluation, and validation of machine learning models for COVID-19 detection based on routine blood tests. Clin. Chem. Lab. Med..

[42] de Moraes Batista A.F., Miraglia J.L., Donato T.H.R., Chiavegatto Filho A.D.P. (2020). COVID-19 diagnosis prediction in emergency care patients: A machine learning approach. MedRxiv.

[43] Kukar M., Gunčar G., Vovko T., Podnar S., Černelč P., Brvar M., Zalaznik M., Notar M., Moškon S., Notar M. (2021). COVID-19 diagnosis by routine blood tests using machine learning. Sci. Rep..

[44] Wang D., Hu B., Hu C., Zhu F., Liu X., Zhang J., Wang B., Xiang H., Cheng Z., Xiong Y. (2020). Clinical characteristics of 138 hospitalized patients with 2019 novel coronavirus–infected pneumonia in Wuhan, China. JAMA.

[45] Chen N., Zhou M., Dong X., Qu J., Gong F., Han Y., Qiu Y., Wang J., Liu Y., Wei Y. (2020). Epidemiological and clinical characteristics of 99 cases of 2019 novel coronavirus pneumonia in Wuhan, China: A descriptive study. Lancet.

[46] Zhang C., Shi L., Wang F.S. (2020). Liver injury in COVID-19: Management and challenges. Lancet Gastroenterol. Hepatol..

[47] Lippi G., Plebani M. (2020). Laboratory abnormalities in patients with COVID-2019 infection. Clin. Chem. Lab. Med..

[48] Kadri F., Dairi A., Harrou F., Sun Y. (2022). Towards accurate prediction of patient length of stay at emergency department: A GAN-driven deep learning framework. J. Ambient. Intell. Humaniz. Comput..

